# Acupuncture effects of post-stroke thalamic pain: a systematic review and meta-analysis of randomized controlled trials

**DOI:** 10.3389/fneur.2025.1528956

**Published:** 2025-04-30

**Authors:** Tianwei Zhang, Junying Zhai, Ling Cheng, Kaixin Jiang, Dayang Wang, Huawei Shi, Bin Wang, Xing Chen, Xinglu Dong, Li Zhou

**Affiliations:** ^1^Dongzhimen Hospital, Beijing University of Chinese Medicine, Beijing, China; ^2^Beijing University of Chinese Medicine, Beijing, China

**Keywords:** acupuncture, stroke, thalamic pain, systematic review, meta-analysis

## Abstract

**Background:**

Post-stroke thalamic pain (PS-TP), a common form of central pain, is characterized by hyperalgesia and abnormal sensations in the contralateral affected area. Acupuncture treatment has shown increasing promise in treating PS-TP in recent years. This systematic review and meta-analysis aimed to evaluate the efficacy and safety of acupuncture treatment for PS-TP.

**Methods:**

According to the established search strategy, randomized controlled trials (RCTs) of acupuncture therapy for PS-TP were retrieved from eight Chinese and English databases as well as two clinical trial registration platforms, up to February 2024. Outcome measures included the total efficacy rate, visual analogue scale (VAS), present pain intensity score (PPI), pain rating index (PRI), β-endorphin (β-EP), substance P (SP) and adverse reactions. Sensitivity analysis and subgroup analysis were conducted to identify the sources of heterogeneity. We evaluated the evidence quality of outcomes via the Grading of Recommendations Assessment, Development, and Evaluation (GRADE) rating system and performed trial sequential analyses using TSA software.

**Results:**

The final inclusion comprised 12 articles, which involved 953 patients. Meta-analysis results indicated that acupuncture treatment for PS-TP was more effective than conventional medical treatment in reducing VAS scores [MD = −1.11, 95% CI (−1.33, −0.88), p = 0.002], PPI scores [MD = −0.65, 95% CI (−1.13, −0.16), p = 0.009], and PRI scores [MD = −1.02, 95% CI (−1.41, −0.63), p < 0.00001]. Additionally, acupuncture treatment for PS-TP was superior to the conventional medical treatment in increasing plasma β-EP levels [MD = 8.83, 95% CI (5.42, 12.25), p < 0.00001], and reducing SP levels [MD = −4.75, 95% CI (−7.11, −2.40), p < 0.0001]. Regarding the total efficacy rate, acupuncture treatment was superior to the conventional medical treatment in treating PS-TP [RR = 1.24, 95% CI (1.17, 1.31), p < 0.00001]. The incidence of adverse events was lower in acupuncture treatment than in conventional medical treatment [RR = 0.43, 95% CI (0.14, 1.32), p = 0.03]. The GRADE assessment indicated that the quality of evidence for all outcome measures ranged from moderate to very low. Trial sequential analysis (TSA) results provided compelling evidence for the efficacy of acupuncture in treating PS-TP.

**Conclusion:**

Acupuncture treatment emerges as a potentially efficacious and safe treatment option for PS-TP. In the future, more large-sample, high-quality RCTs are needed to provide primarily high-level evidence in evidence-based medicine regarding the safety and sustained effects of acupuncture treatment for PS-TP.

**Systematic review registration:**

https://www.crd.york.ac.uk/PROSPERO/view/CRD42024498698, identifier CRD42024498698.

## 1 Introduction

Post-stroke thalamic pain (PS-TP), a common form of central pain, is characterized by hyperalgesia and abnormal sensations in the contralateral affected area. In many patients, this pain tends to progressively worsen, significantly affecting their quality of life and psychological well-being (1). Modern medicine recognizes that pain, along with the anxiety and depression it induced, constitutes a cycle of negative emotions that can intensify the perception of pain (2). In some cases, these psychological states may further aggravate the pain. The pathogenesis of PS-TP involves spinothalamic tract dysfunction and neuroinflammatory cascades. Central sensitization arises from loss of inhibitory regulation in the lateral tract and hyperexcitability in the medial tract, driven by compensatory neural hyperactivity post-stroke (3). Neuroglial remodeling further exacerbates pain through microglial activation, which releases signaling molecules (e.g., SDF1, ITC) to trigger cytokine cascades and pro-inflammatory mediators (IL-1β, IL-6β), disrupting central pain modulation and sustaining nociception (4).

Despite these advances in understanding its pathophysiology, there are currently few effective medications available that directly target the underlying causes of PS-TP. Some domestic and international treatment guidelines recommend antidepressants, antiepileptic drugs, opioid analgesics, and other medications to alleviate symptoms. Non-pharmacological interventions, such as repetitive transcranial magnetic stimulation, electrical stimulation, and deep brain stimulation, are also suggested (5). However, these approaches often fall short in efficacy and fail to achieve the desired therapeutic outcomes (6).

In contrast to these conventional approaches, traditional Chinese medicine (TCM) has shown increasing promise in treating PS-TP in recent years. Multiple clinical trials have validated that TCM, particularly acupuncture, offers more significant therapeutic effects and higher safety than conventional drugs and non-pharmacological interventions (7–9). Acupuncture, a cornerstone of TCM, is valued for its ability to address multiple symptoms, targets, and mechanisms. Beyond pain modulation, TCM also posits that acupuncture can help regulate emotions and alleviate the negative psychological states associated with PS-TP.

Despite accumulating empirical support, existing evidence remains fragmented due to small sample sizes, heterogeneous methodologies, and insufficient attention to long-term efficacy. Moreover, existing systematic reviews have not adequately assessed the methodological quality of evidence and robustness of the findings. To address this gap, we conducted this study to systematically evaluate the efficacy and safety of acupuncture therapy compared to conventional medical treatment for PS-TP. The aim of the study was to generate evidence-based recommendations and inform future clinical guideline development for PS-TP management.

## 2 Subject/materials and methods

### 2.1 Method

The PROSPERO International Prospective Register of Systematic Reviews had an agreement for this meta-analysis registered under registration number CRD42024498698. The Preferred Reporting Items for Systematic Reviews and Meta-Analyses (PRISMA) standards were adhered to in the preparation of the report (10).

#### 2.1.1 Search strategy

In addition to two clinical trial registries, ClinicalTrials.gov and the Chinese Clinical Trial Registry, we also searched eight English and Chinese databases: PubMed, MEDLINE (via Embase), Cochrane Library, Embase, China National Knowledge Infrastructure (CNKI), China Biomedical Literature Database (CBM), VIP Database, and Wanfang Data. This search covered randomized controlled trials (RCTs) published from the inception of these databases up to February 2024. Different combinations of Medical Subject Headings (MeSH) and non-MeSH phrases were used in the search, including “thalamic Diseases,” “thalamic pain,” “thalami syndromes,” “stroke,” “cerebrovascular accident,” “acupuncture,” “acupuncture therapy,” “pharmacopuncture,” “pharmacoacupuncture,” “acupuncture treatment,” “randomized” and “randomized controlled trial.” No restrictions were placed on language, study population, or country. Table 1 provides specific search technique details.

**Table 1:** Search strategy for PubMed.

Number	Search terms
#1	“Thalamic Diseases”[Mesh]
#2	“Disease, Thalamic”[Title/Abstract] OR “Diseases, Thalamic”[Title/Abstract] OR “Thalamic Disease”[Title/Abstract] OR “Dejerine-Roussy Syndrome”[Title/Abstract] OR “Dejerine Roussy Syndrome”[Title/Abstract] OR “Syndrome, Dejerine-Roussy”[Title/Abstract] OR “Thalamic Syndrome”[Title/Abstract] OR “Syndrome, Thalamic”[Title/Abstract] OR “Syndromes, Thalamic”[Title/Abstract] OR “Thalamic Syndromes”[Title/Abstract] OR “thalamic pain”[Title/Abstract] OR “thalamic pain syndrome”[Title/Abstract]
#3	#1 or #2
#4	“Stroke”[Mesh]
#5	“Strokes”[Title/Abstract] OR “Cerebrovascular Accident”[Title/Abstract] OR “Cerebrovascular Accidents”[Title/Abstract] OR “CVA (Cerebrovascular Accident)”[Title/Abstract] OR “CVAs (Cerebrovascular Accident)”[Title/Abstract] OR “Cerebrovascular Apoplexy”[Title/Abstract] OR “Apoplexy, Cerebrovascular”[Title/Abstract] OR “Vascular Accident, Brain”[Title/Abstract] OR “Brain Vascular Accident”[Title/Abstract] OR “Brain Vascular Accidents”[Title/Abstract] OR “Vascular Accidents, Brain”[Title/Abstract] OR “Cerebrovascular Stroke”[Title/Abstract] OR “Cerebrovascular Strokes”[Title/Abstract] OR “Stroke, Cerebrovascular”[Title/Abstract] OR “Strokes, Cerebrovascular”[Title/Abstract] OR “Apoplexy”[Title/Abstract] OR “Cerebral Stroke”[Title/Abstract] OR “Cerebral Strokes”[Title/Abstract] OR “Stroke, Cerebral”[Title/Abstract] OR “Strokes, Cerebral”[Title/Abstract] OR “Stroke, Acute”[Title/Abstract] OR “Acute Stroke”[Title/Abstract] OR “Acute Strokes”[Title/Abstract] OR “Strokes, Acute”[Title/Abstract] OR “Cerebrovascular Accident, Acute”[Title/Abstract] OR “Acute Cerebrovascular Accident”[Title/Abstract] OR “Acute Cerebrovascular Accidents”[Title/Abstract] OR “Cerebrovascular Accidents, Acute”[Title/Abstract]
#6	#4 or #5
#7	“Acupuncture”[Mesh] OR “Acupuncture Therapy”[Mesh]
#8	“Acupuncture Treatment”[Title/Abstract] OR “Acupuncture Treatments”[Title/Abstract] OR “Treatment, Acupuncture”[Title/Abstract] OR “Therapy, Acupuncture”[Title/Abstract] OR “Pharmacoacupuncture Treatment”[Title/Abstract] OR “Treatment, Pharmacoacupuncture”[Title/Abstract] OR “Pharmacoacupuncture Therapy”[Title/Abstract] OR “Therapy, Pharmacoacupuncture”[Title/Abstract] OR “Acupotomy”[Title/Abstract] OR “Acupotomies”[Title/Abstract] OR “Pharmacopuncture”[Title/Abstract]
#9	#7 or #8
#10	“randomized controlled trial”[Publication Type] OR “randomized”[Title/Abstract] OR “placebo”[Title/Abstract]
#11	#3 and #6 and #9 and #10

#### 2.1.2 Inclusion criteria

(1) All RCTs published in Chinese or English that had an eligible intervention and/or outcome for PS-TP were included. The included RCTs should adhere carefully to the randomization principle; nevertheless, there were no stringent restrictions because blinding and placebo control in acupuncture therapy are challenging to adhere to.
(2) The subjects that were included had to fulfill the PS-TP diagnostic requirements.
(3) Acupuncture was administered to the experimental groups either in addition to or instead of medication, and pharmaceuticals were used as the control group. Body acupuncture, scalp acupuncture, plum-blossom acupuncture, three-edged acupuncture, electro-acupuncture, wrist-ankle acupuncture were all included.
(4) Outcome indicators: At least one obvious indicator such as the total efficacy rate, visual analogue scale (VAS), present pain intensity score (PPI), pain rating index (PRI), β-endorphin (β-EP), substance P (SP) and adverse reactions was included.

#### 2.1.3 Exclusion criteria

(1) Studies that did not account for the type of study.
(2) Research that used non-drug treatment in the control group or non-acupuncture treatment in the treatment group were rejected.
(3) Studies were excluded if repeated attempts to contact the authors failed to retrieve the full text or accessible data.
(4) Repeated publications.

### 2.2 Studies selection process

**Figure 1:**
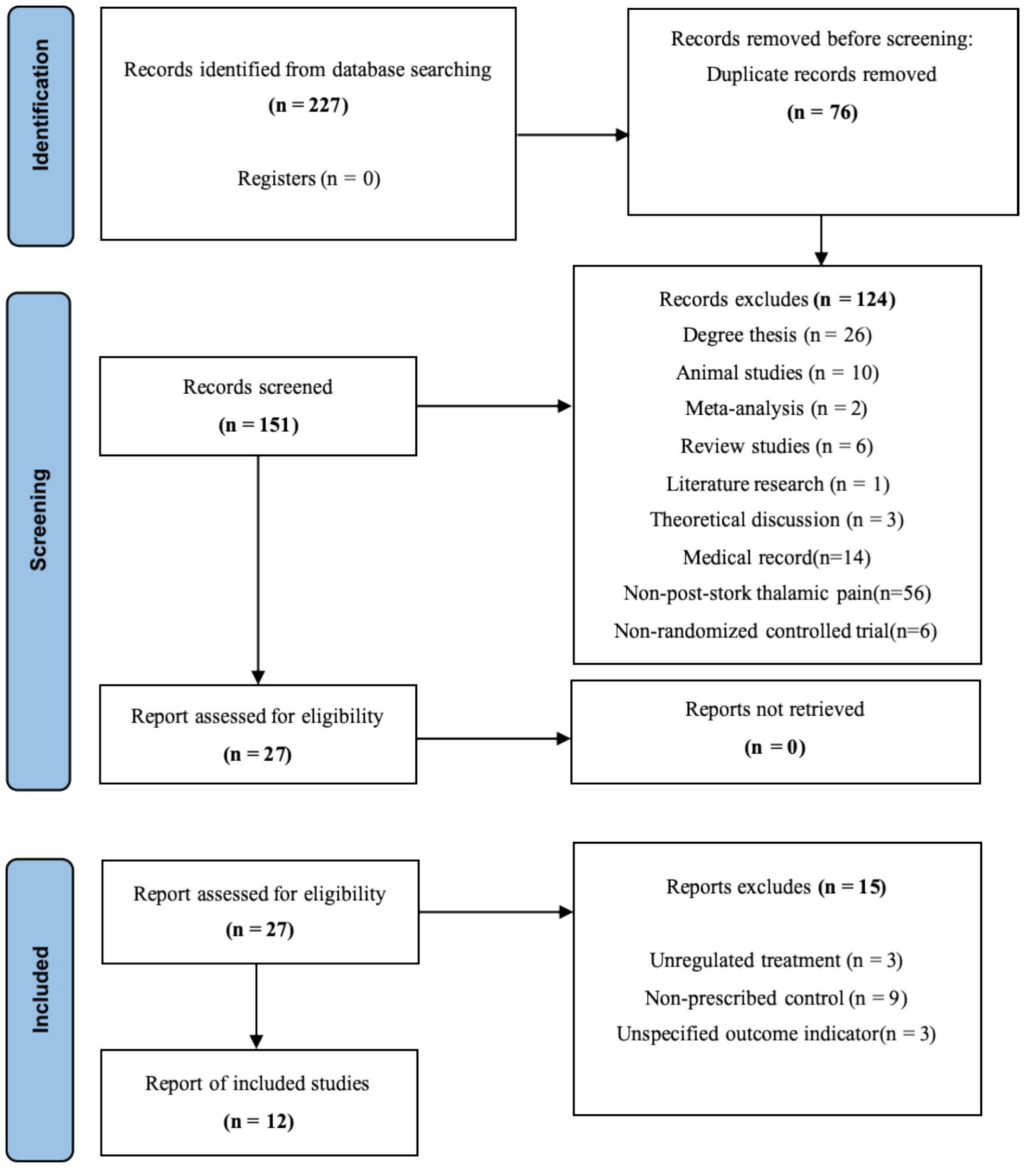
Literature screening flowchart.

Figure 1 illustrates the selection procedure for research. First, duplicate articles were eliminated from the obtained literature by using EndNote X9 was automatic review feature. Second, unidentified duplicates, such as reports on different facets of the same research as well as duplicates from several journals and multilingual publications, were sorted out by two independent investigators (TZ and LC). After that, these two researchers looked through the article titles and abstracts to choose relevant research studies according to the kind of study, the interventions/comparators, and the results. Thirdly, two investigators (TZ and LC) conducted a full-text assessment in order to omit publications based on the specified exclusion criteria. Any disagreements were settled by consensus or after speaking with DXL, a third investigator.

### 2.3 Risk of bias assessment

The Cochrane scoring system was used to gauge the methodological quality of trials that satisfied eligibility criteria (11). Based on seven criteria, including random sequence generation methodology, treatment allocation concealment, participant, healthcare provider, data collector, outcome assessor, and data analyst blinding, infrequently missing outcome data, and absence of selective outcome reporting, each included study was assessed by two independent researchers (TZ and KJ). One of the three categories—low risk, high risk, or unclear risk of bias—was assigned to the evaluation. After the study assessment was cross-checked, a third investigator (XD) determined any disagreements with the protocols.

### 2.4 Data extraction

Two researchers (KJ and JZ) independently retrieved data from studies that qualified. Data about the study (authors, publication year, design, follow-up period, etc.); participant characteristics (age, gender, duration, diagnosis, etc.); intervention/control method (number of treatments, frequency, etc.); and outcomes (types of primary and secondary outcome measures, the total efficacy rate, mean and standard deviation (SD) of VAS scores, PPI scores, PRI scores, adverse event, etc.) were all entered into an electronic data-extraction form.

### 2.5 Data synthesis and statistical analysis

We conducted all analyses using Review Manager (version 5.4), Stata (version 14.0), and TSA 0.9.5.10 Beta. Specifically, meta-analyses were performed with Review Manager, trial sequential analyses were conducted using TSA software, and publication bias was assessed through Stata. Heterogeneity was investigated in the involved publications. A fixed-effects model was employed when *p* ≥ 0.05 or *I*^2^ < 50% indicated that the heterogeneity among the studies was deemed to be minimal; however, when *p* < 0.05 or *I*^2^ ≥ 50% suggested that there was heterogeneity between studies, a random-effects model was employed. When appropriate, sensitivity analyses could be performed to examine the sources of heterogeneity. For the measurement data, the relative risk (RR) was utilized, and for the count data, the mean difference (MD). There was a 95% confidence interval (CI) attached to both. The difference was statistically significant when *p* ≤ 0.05. The quality of evidence was assessed using the Grading of Recommendations Assessment, Development, and Evaluation (GRADE) rating system approach. The evidence quality was categorized into four levels: high, moderate, low, and very low. Two reviewers (TZ and HS) independently assessed the certainty of evidence using GRADE criteria. Discrepancies were resolved by consensus.

## 3 Results

### 3.1 Study selection

Initial database searching identified 227 distinct records, as seen in Figure 1. Seventy-six of these studies were disqualified for being duplicated. The following criteria led to the exclusion of an additional 139 studies: degree thesis (*n* = 26), animal studies (*n* = 10), meta-analyses (*n* = 2), review studies (*n* = 6), literature research (*n* = 1), theoretical discussions (*n* = 3), medical records (*n* = 14), non-PS-TP (*n* = 56), non-RCTs (*n* = 6), unregulated treatments (*n* = 3), non-prescribed controls (*n* = 9), and unspecified outcome indicators (*n* = 3). Ultimately, 12 studies were included in the analysis.

### 3.2 Characteristics of the included studies

The characteristics of the 12 included studies (12–23) with 953 subjects are detailed in Table 2. All studies were conducted in China and published between 2014 and 2023. Sample sizes ranged from 26 to 200, with treatment duration varying from 14 to 60 days. All participants were diagnosed with PS-TP. Four studies included patients with ischemic stroke, while eight studies did not specify the stroke type, encompassing both ischemic and hemorrhagic strokes. Four studies included period with non-acute, while eight studies did not specify the period. Gender and average age data showed no statistically significant differences, ensuring comparability. Of the included studies, five compared manual acupuncture (MA) to Western medicine (WM), five compared MA plus TCM with WM, one compared MA plus WM with WM, and one compared MA plus TCM plus WM with WM. The most frequently used MA was Xingnao Kaiqiao acupuncture, and the most common WM was Carbamazepine. The 12 studies provided data on 12 comparisons for the total efficacy rate, 12 comparisons for VAS, five comparisons for PPI and PRI, three comparisons for β-EP, two comparisons for SP, two comparisons for aftereffects, and five comparisons for adverse event rates.

**Table 2:** The basic characteristics of studies included.

	Stroke type	Period		Gender (male/female)		Average age		Intervention		Course of treatment	Follow-up	Outcomes
Study ID			Sample size (T/C)	T	C	T	C	T	C			
Chao Y. (2021)	Ischemic stroke	Non-acute	33/34	19/14	21/13	60.58 ± 8.09	60.32 ± 7.32	MA + TCM	Carbamazepine	28 days	NA	①②③④
Kong Y. (2018)	Unclear	Unclear	21/21	18/3	13/8	64.30 ± 12.31	63.42 ± 11.26	MA	Pregabalin	T14/C 28 days	14d after treatment	①②③④⑤
Li Y. J. (2017)	Unclear	Unclear	32/32	20/12	18/14	55 ± 9	58 ± 8	MA	Pregabalin	56 days	90d after treatment	①②③④⑤⑥
Lu M. (2018)	Unclear	Unclear	40/40	32/8	30/10	55.25 ± 4.39	59.43 ± 5.82	MA	Carbamazepine	28 days	NA	①②③④
Lu Y. (2021)	ischemic stroke	Non-acute	56/56	31/25	30/26	59.16 ± 9.25	57.81 ± 8.73	MA + TCM	Amitriptyline	28 days	NA	①②⑦
Wang M. (2014)	ischemic stroke	Unclear	42/42	30/12	28/14	62.6	60.4	MA + TCM	Carbamazepine	28 days	NA	①②⑦
Wang S. M. (2018)	Unclear	Unclear	30/30	14/16	15/15	55.4 ± 6.6	57.3 ± 5.8	MA	Pregabalin	60 days	NA	①②⑦
Yang X. H. (2022)	Unclear	Non-acute	30/30	16/14	17/13	60.75 ± 4.26	60.82 ± 4.15	MA + TCM	Carbamazepine	30 days	NA	①②③④⑤⑥
Ying D. S. (2016)	Unclear	Unclear	31/31	23/8	21/10	59.65 ± 7.01	59.96 ± 6.95	MA + TCM	Carbamazepine	28 days	NA	①②
Zhang Q. X (2021)	ischemic stroke	Non-acute	48/48	27/21	28/20	62.71 ± 10.14	61.28 ± 9.56	MA + TCM + Carbamazepine	Carbamazepine	28 days	NA	①②⑦
Zheng W. F. (2023)	Unclear	Unclear	100/100	59/41	58/42	53.50 ± 3.15	53.00 ± 3.12	MA + pregabalin	Pregabalin	56 days	NA	①②
Zhu F. B. (2014)	Unclear	Unclear	13/13	7/6	7/6	63.21 ± 1.96	63.12 ± 2.74	MA	Carbamazepine	14 days	NA	①②⑦

Outcomes: ① = the total efficiency, ② = VAS, ③ = PPI, ④ = PRI, ⑤ = β-EP, ⑥ = SP, ⑦ = AE. C, control group; MA, manual acupuncture; T, treatment group; TCM, traditional Chinese medicine.

### 3.3 Risk of bias

**Figure 2:**
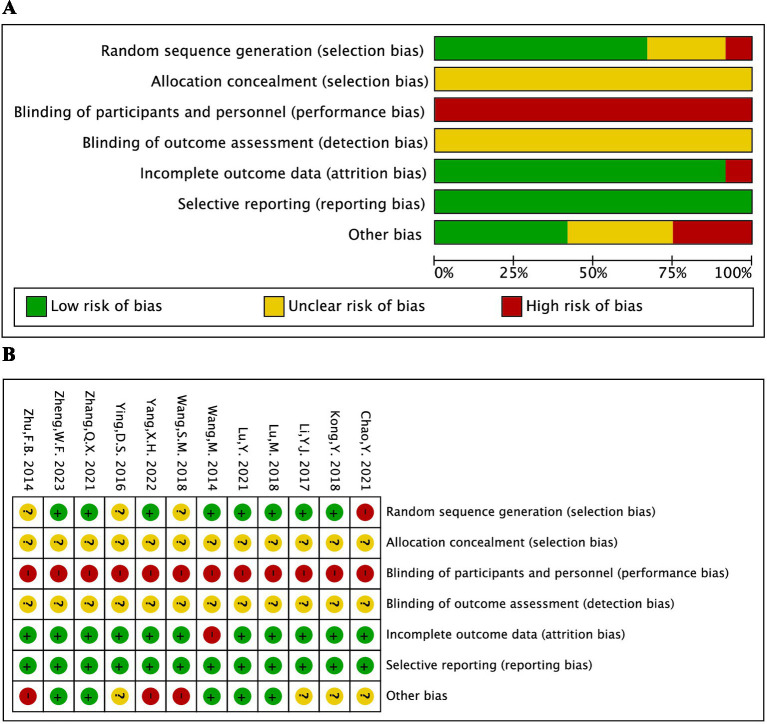
Risk-of-bias summary **(A)** and graph **(B)** were summarized for all included studies.

The risk of bias evaluation for each study is shown in Figure 2. The results indicated that only one study, which used incorrect methods for random sequence generation, was identified as high risk. Additionally, three studies did not specify the randomization methods. None of the 12 studies reported allocation concealment or blinded participants, personnel, or outcome assessors. One study had 12 participants lost to follow-up or excluded, while the remaining 11 studies had complete data. All 12 studies provided detailed reports on all outcome indicators. Three studies had only one author each, and four did not adequately describe other potential risk factors.

### 3.4 Meta-analysis results

#### 3.4.1 VAS

**Figure 3:**
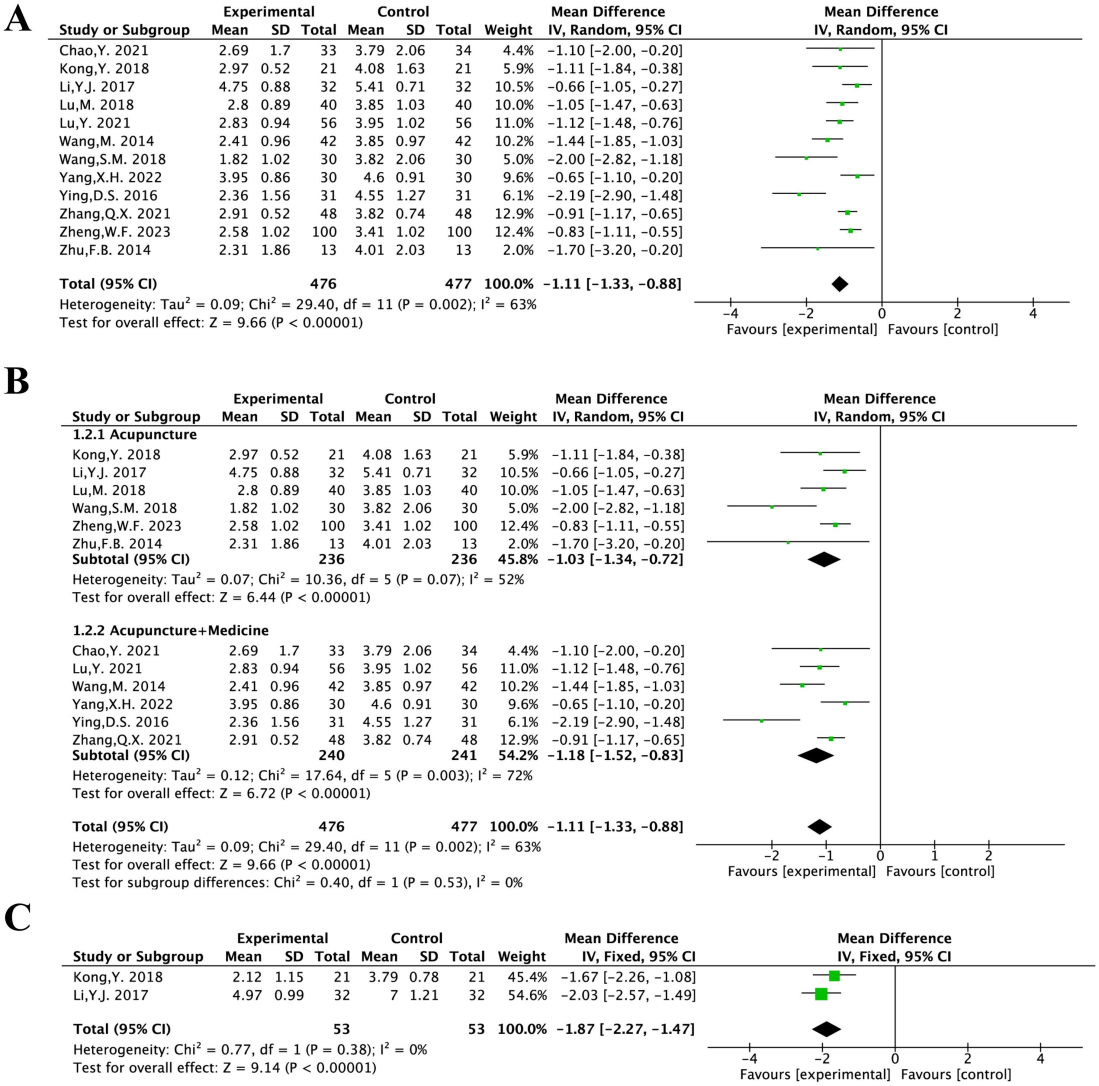
The forest plot of the VAS score **(A)** based on the difference in intervention methods **(B)** and aftereffect **(C)**.

The VAS score was evaluated in 12 included studies and covered 953 patients (12–23). Following heterogeneity testing (*I*^2^ = 63%, *p* = 0.002), it suggested statistically significant heterogeneity among the selected literature in this study, requiring exploration of heterogeneity (Figure 3A).

Sensitivity analysis to find sources of heterogeneity: In the sensitivity analysis of the 12 included studies, it was found that the survey by Ying D. S. 2016 had a significant impact on heterogeneity (13). This study was a single-centre RCT and the findings may be attributed to the relatively significant efficacy observed in the study combined with its small sample size. Therefore, a random-effects model was employed for the meta-analysis of 12 studies.

This finding suggested that acupuncture treatment for PS-TP reduces VAS scores more effectively than the conventional medical treatment [MD = −1.11, 95% CI (−1.33, −0.88), *p* = 0.002].

Based on the intervention methods, the 12 articles were categorized into “MA” and “MA + TCM” groups for subgroup analysis.

Heterogeneity testing was performed on the MA group; results showed that there was significant and statistically meaningful heterogeneity among the included studies (*I*^2^ = 52%, *p* = 0.07). A random-effects model was therefore used. It was statistically significant [MD = −1.03, 95% CI (−1.34, −0.72), *p* < 0.00001] and showed that acupuncture treatment was more effective than the conventional medical treatment in improving VAS scores for PS-TP.

The MA + TCM group was heterogeneity testing (*I*^2^ = 72%, *p* = 0.003), indicating a large degree of statistically significant heterogeneity among the chosen studies. Differences in the kinds of Chinese herbal formulations utilized in addition to acupuncture could be the cause of the notable variation. A random-effects model was therefore used. The results showed that acupuncture in conjunction with TCM was more effective than the conventional medical treatment in improving VAS scores for PS-TP. It was statistically significant [MD = −1.18, 95% CI (−1.52, −0.83), *p* < 0.00001] (Figure 3B).

Two literatures included in this study conducted aftereffect evaluations of VAS (14, 17). There was no substantial heterogeneity among the chosen literatures, according to the results of the heterogeneity test (*I*^2^ = 0%, *p* = 0.38). For meta-analysis, a fixed-effects model could therefore be applied.

This difference was statistically significant, suggesting that acupuncture treatment was more effective than the conventional medical treatment in reducing VAS scores aftereffect for PS-TP [MD = −1.87, 95% CI (−2.27, −1.47), *p* < 0.00001] (Figure 3C).

#### 3.4.2 PPI

The PPI was evaluated in five included studies and covered 313 patients (14, 15, 17, 20, 21). After heterogeneity testing (*I*^2^ = 95%, *p* < 0.00001), showing a considerable degree of heterogeneity in the chosen literature. Thus, for the meta-analysis, a random-effects model was employed.

**Figure 4:**
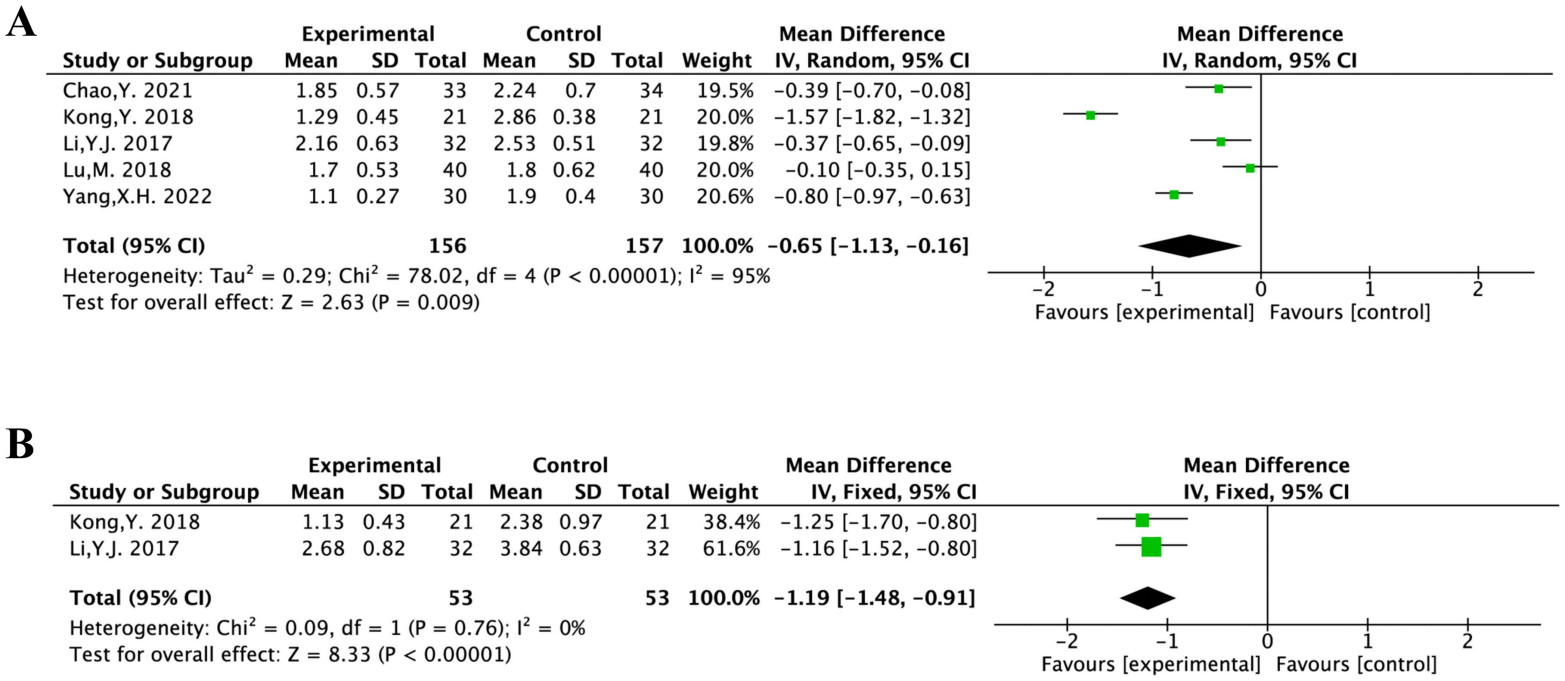
The forest plot of the PPI score **(A)** and aftereffect **(B)**.

It was statistically significant, suggesting that acupuncture treatment was more effective than the conventional medical treatment in reducing PPI scores for PS-TP [MD = −0.65, 95% CI (−1.13, −0.16), *p* = 0.009] (Figure 4A).

Two studies included in this study conducted aftereffect evaluations of PPI (14, 17). There was no substantial heterogeneity among the chosen literature, according to the results of the heterogeneity test (*I*^2^ = 0%, *p* = 0.76). For meta-analysis, a fixed-effects model could therefore be applied.

It was statistically significant, suggesting that acupuncture treatment was more effective than the conventional medical treatment in reducing PPI scores for PS-TP [MD = −1.19, 95% CI (−1.48, −0.91), *p* < 0.00001] (Figure 4B).

#### 3.4.3 PRI

The PRI was evaluated in five included studies and covered 953 patients (14, 15, 17, 20, 21). The heterogeneity testing (*I*^2^ = 76%, *p* = 0.002) showed a considerable degree of heterogeneity in the chosen literature. Based on the intervention methods, the five articles were categorized into “MA” and “MA + TCM” groups for subgroup analysis.

**Figure 5:**
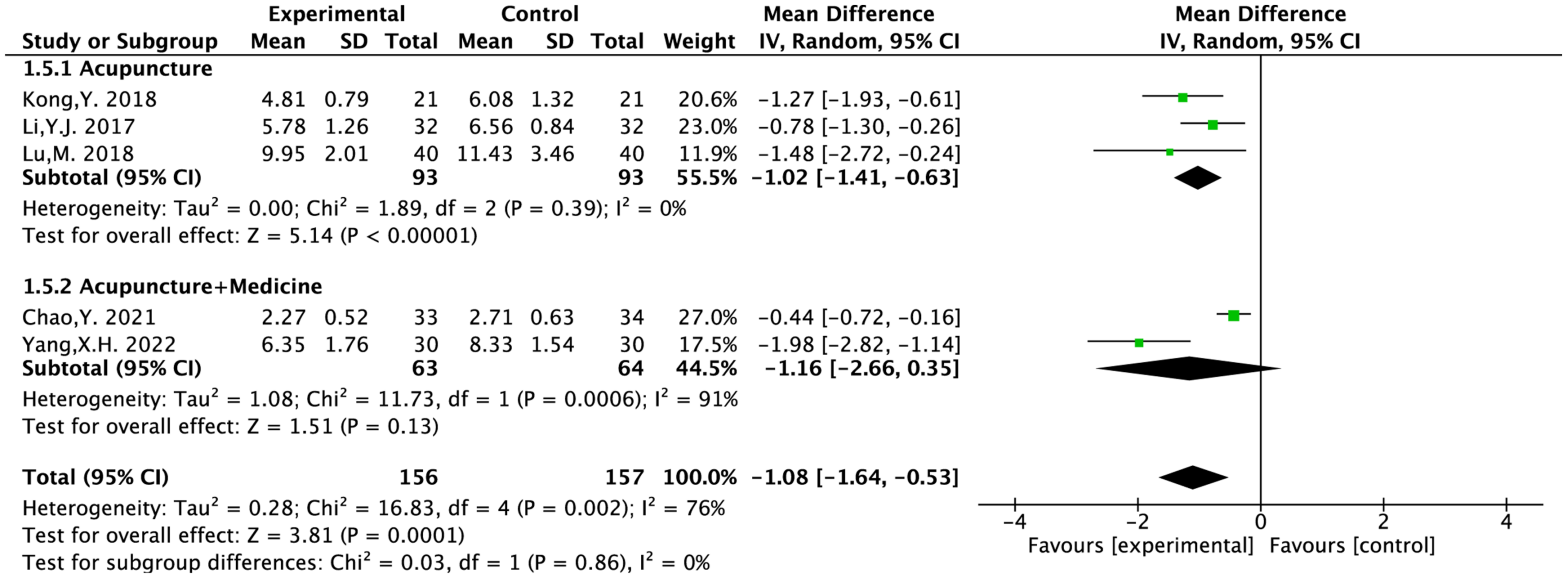
The forest plot of the PRI score based on the difference in intervention methods.

The MA group was tested for heterogeneity (*I*^2^ = 0%, *p* = 0.39) showing no significant heterogeneity among the included studies. It was statistically significant [MD = −1.02, 95% CI (−1.41, −0.63), *p* < 0.00001]. The MA + TCM group showed significant heterogeneity (*I*^2^ = 91%, *p* = 0.0006). Analysis results indicated that acupuncture therapy for PS-TP was superior to the conventional medical treatment in reducing PRI scores (Figure 5).

Two articles evaluated PRI for aftereffect (14, 17). The heterogeneity testing (*I*^2^ = 98%, *p* < 0.00001) showed a considerable degree of heterogeneity in the chosen literature and considered that the acupuncture frequency and duration were different in the two studies: twice daily for 2 weeks and 6 days per week for 8 weeks. The high heterogeneity might be attributable to the differing frequencies and durations. Given the non-overlapping confidence intervals between the two studies and substantial heterogeneity (*I*^2^ > 75%), a meta-analysis was deemed inappropriate. The results of each study were therefore presented separately with a narrative synthesis.

The MD of Li Y. J. 2017 was −4.66 with a 95% CI of (−5.29, −4.03). The MD of Kong Y. 2018 was −1.54 with a 95% CI of (−1.98, −1.10). Both studies suggested that acupuncture was superior to standard pharmacotherapy in alleviating PS-TP. Both studies reported that acupuncture treatment was more effective than the conventional medical treatment in reducing PRI scores for PS-TP.

#### 3.4.4 β-EP

The β-EP was evaluated in three included studies and covered 166 patients (14, 17, 21). Following heterogeneity testing (*I*^2^ = 70%, *p* = 0.04), it indicated that there was statistically significant variation in the chosen literature. For the meta-analysis, a random-effects model was thus selected.

**Figure 6:**
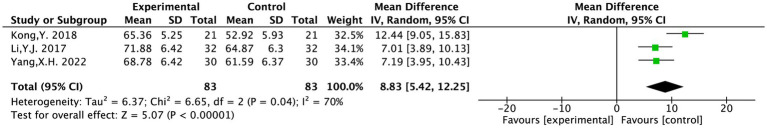
The forest plot of the β-EP.

The findings suggested that acupuncture treatment for PS-TP was superior to the conventional medical treatment in increasing plasma β-EP levels [MD = 8.83, 95% CI (5.42, 12.25), *p* < 0.00001] (Figure 6).

#### 3.4.5 SP

The SP was evaluated in two included studies and covered 124 patients (14, 21). The heterogeneity testing (*I*^2^ = 0%, *p* = 0.96) showed no significant heterogeneity among the included studies. Therefore, a fixed-effects model could be used for meta-analysis.

**Figure 7:**
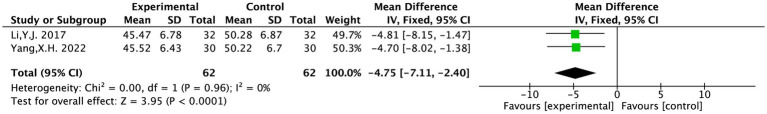
The forest plot of the SP.

The results implied that the conventional medical treatment’s SP levels were not as effectively reduced by acupuncture treatment [MD = −4.75, 95% CI (−7.11, −2.40), *p* < 0.0001] (Figure 7).

#### 3.4.6 The total efficacy rate

The 12 articles included in this study underwent testing for heterogeneity (*I*^2^ = 32%, *p* = 0.14). It suggested that there was no statistically significant heterogeneity across the chosen literature. Thus, a fixed-effects model was chosen for the meta-analysis.

**Figure 8:**
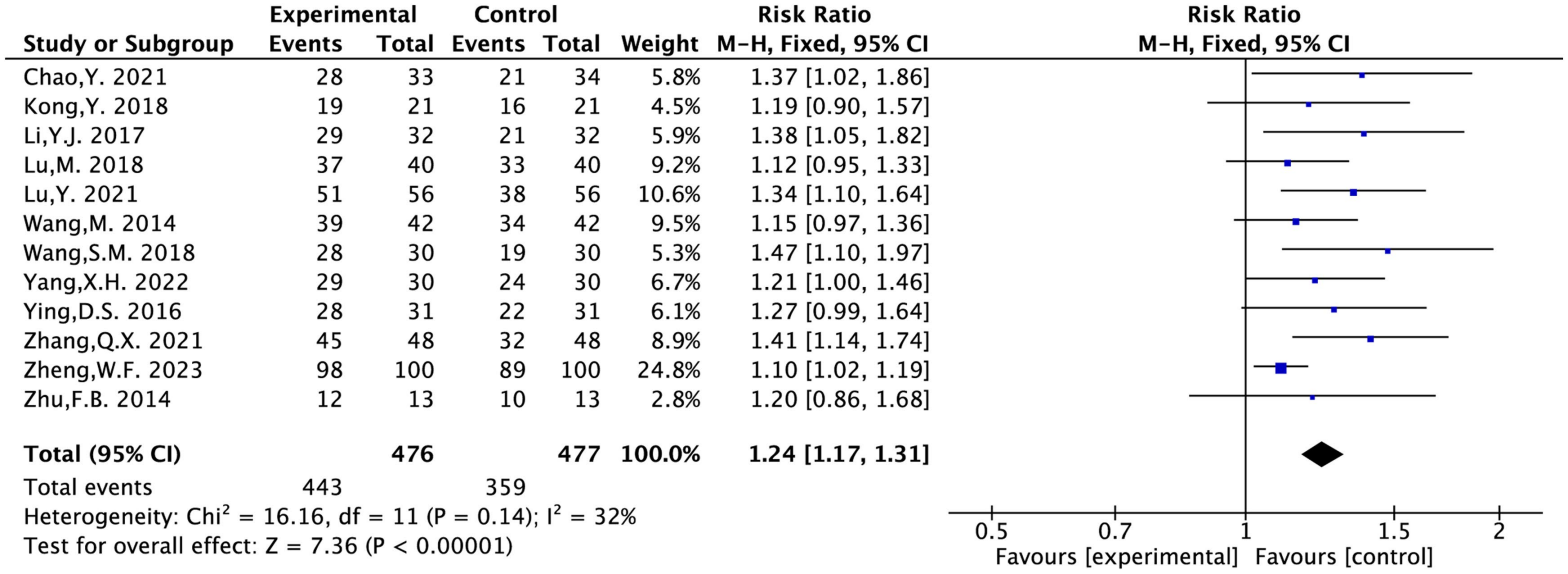
The forest plot of the total efficacy rate.

The result indicated that acupuncture was superior to the conventional medical treatment in treating PS-TP [RR = 1.24, 95% CI (1.17, 1.31), *p* < 0.00001] (Figure 8).

#### 3.4.7 Safety evaluation

The rate of AE was evaluated in five included studies and covered 378 patients (12, 16, 18, 19, 23). Following heterogeneity testing (*I*^2^ = 62%, *p* = 0.03), it indicated statistically significant heterogeneity between the chosen literature in this study, requiring exploration of heterogeneity.

Sensitivity analysis to locate sources of heterogeneity: In a sensitivity analysis of the five included studies, Zhang Q. X. 2021 was found to exert a significant influence on heterogeneity (18). It was considered that the heterogeneity might be due to this study recording adverse reactions such as dizziness, dry mouth, and bruising after acupuncture, which differed from the adverse reaction contents recorded in other studies. A random-effects model was therefore employed.

**Figure 9:**
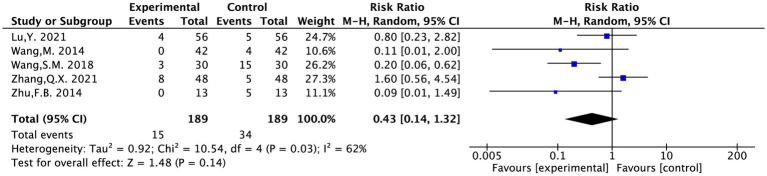
The forest plot of the adverse event rate.

The results suggested that acupuncture treatment for PT-SP was safer than the conventional medical treatment [RR = 0.43, 95% CI (0.14, 1.32), *p* = 0.03] (Figure 9).

### 3.5 Publication bias detection

**Figure 10:**
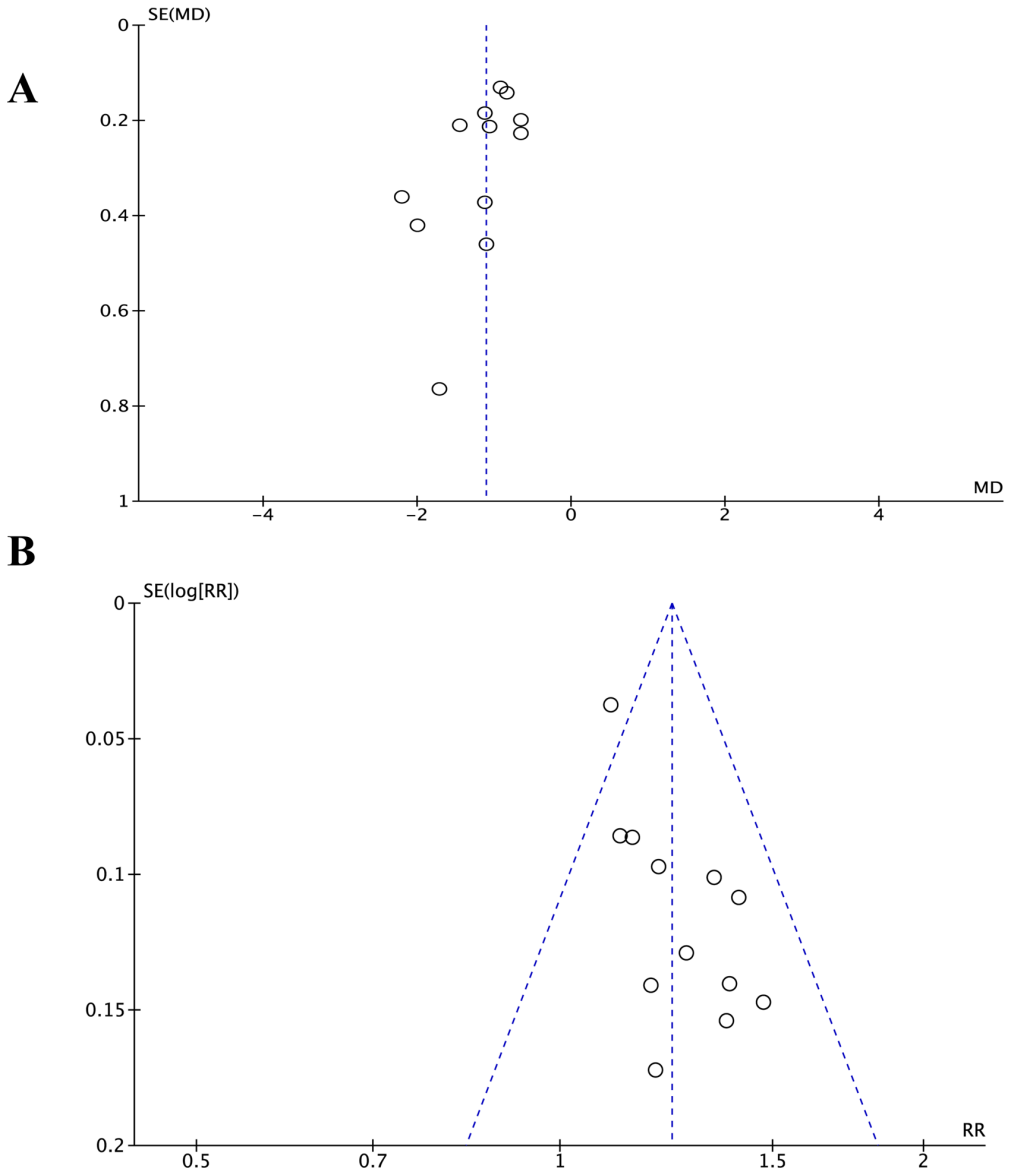
Funnel plot for the VAS **(A)** and total efficacy rate **(B)**.

Investigating publication bias using funnel plots of VAS scores and the total efficacy rate, both showed mild asymmetry. Egger’s test results for VAS scores (*p* = 0.075) and for the total efficacy rate (*p* = 0.532) suggested no publication bias (Figure 10).

### 3.6 GRADE assessment

The GRADE system was utilized to evaluate the overall quality of the evidence. For all outcome measures, the evidence quality ranged from moderate to very low. The primary factor contributing to this decline in quality was the lack of blinding. Additional concerns included high heterogeneity among the studies, small sample sizes, and unclear allocation concealment (Table 3).

**Table 3:** Summary of quality of evidence for outcomes.

			Quality assessment					
Outcomes	No. of participants (studies)	Effect size (95% CI) quality assessment	Risk of bias	Inconsistency	Indirectness	Imprecision	Other considerations (publication bias)	Quality of evidence
The total efficiency	953 (12 studies)	RR = 1.24 [1.17, 1.31]	Serious^a^	Not serious	Not serious	Not serious	Not serious	㊉㊉㊉㊀ Moderate
VAS	953 (12 studies)	MD = −1.11 [−1.33, −0.88]	Serious^a^	Serious^b^	Not serious	Not serious	Not serious	㊉㊉㊀㊀ Low
VAS (MA)	472 (6 studies)	MD = −1.03 [−1.34, −0.72]	Serious^a^	Serious^b^	Not serious	Not serious	Not serious	㊉㊉㊀㊀ Low
VAS (MA + TCM)	481 (6 studies)	MD = −1.18 [−1.52, −0.83]	Serious^a^	Serious^b^	Not serious	Not serious	Not serious	㊉㊉㊀㊀ Low
PPI	313 (5 studies)	MD = −0.65 [−1.13, −0.16]	Serious^a^	Serious^b^	Not serious	Serious^c^	Unclear^d^	㊉㊀㊀㊀ Very low
PRI	313 (5 studies)	MD = −1.08 [−1.64, −0.53]	Serious^a^	Serious^b^	Not serious	Serious^c^	Unclear^d^	㊉㊀㊀㊀ Very low
β-EP	166 (3 studies)	MD = 8.83 [5.42, 12.25]	Serious^a^	Serious^b^	Not serious	Serious^c^	Unclear^d^	㊉㊀㊀㊀ Very low
SP	124 (2 studies)	MD = −4.75 [−7.11, −2.40]	Serious^a^	Not serious	Not serious	Serious^c^	Unclear^d^	㊉㊀㊀㊀ Very low
Adverse reactions	378 (5 studies)	RR = 0.43 [0.14, 1.32]	Serious^a^	Serious^b^	Not serious	Serious^c^	Unclear^d^	㊉㊀㊀㊀ Very low

a Lack of blinding participants or personnel and allocation concealment.

b Heterogeneity among included studies (*p* < 0.05 or *I*^2^ ≥ 50%).

c The sample size included in the outcome is too small.

d Publication bias is probably.

### 3.7 TSA

**Figure 11:**
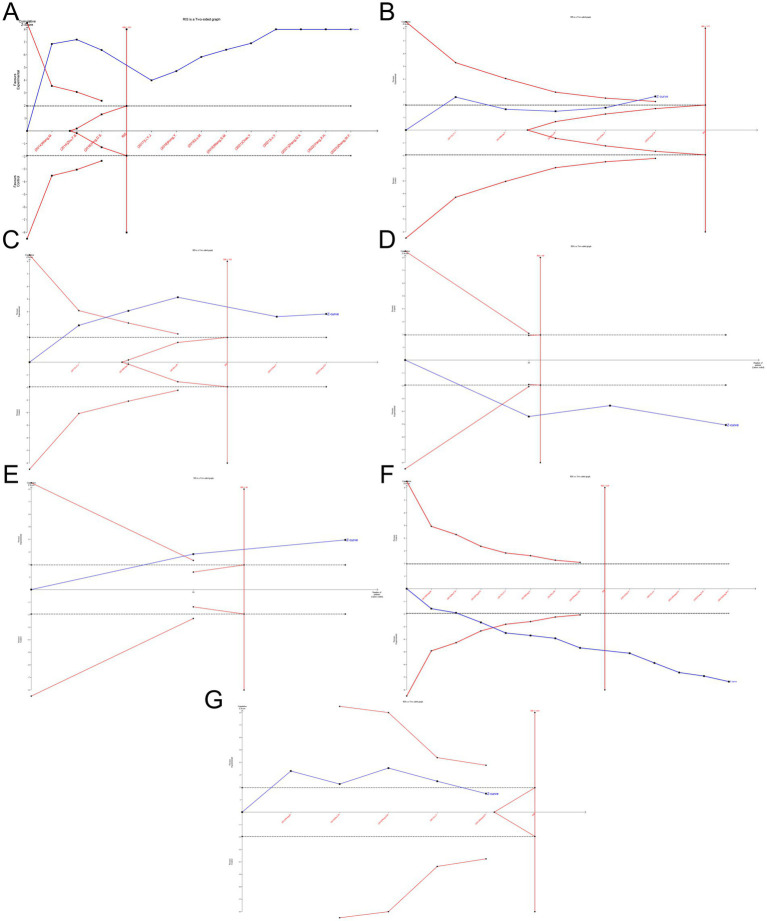
TSA for the VAS **(A)**, PPI **(B)**, PRI **(C)**, β-EP **(D)**, SP **(E)**, total efficacy rate **(F)** and adverse reactions **(G)**.

To estimate the required sample size for the meta-analysis and reduce the risk of false-positive results owing to random error, TSA was conducted for VAS scores, PPI scores, PRI scores, β-EP, SP, total efficacy rates, and adverse reactions. The *Z*-value curves (solid blue lines) for VAS scores, PPI scores, PRI scores, β-EP, SP, and total efficacy rates exceed both the traditional threshold (black dashed line) and the TSA threshold (solid red line). Except for PPI, the cumulative sample sizes for these outcomes had reached the required information size (RIS), allowing for definitive conclusions. Although the cumulative sample size for PPI had not yet reached the RIS, additional trials were not deemed necessary, permitting early definitive conclusions. For adverse reactions, the *Z*-value curves exceed the traditional threshold but did not meet the TSA threshold. The cumulative data had also not reached the expected amount, suggesting that conventional meta-analysis might lead to false-positive results. Therefore, additional trials were needed to confirm these findings (24) (Figure 11).

## 4 Discussion

### 4.1 Main findings

#### 4.1.1 Pain scores

There was significant heterogeneity among pain indicators and subgroups, likely due to the diverse geographical locations and varying acupuncture techniques used in the studies. All 12 clinical trials included in this analysis reported positive outcomes, indicating that acupuncture was more effective than Western medication in reducing VAS, PPI, and PRI scores for PS-TP. Additionally, the combination of acupuncture with medication also showed notable efficacy in reducing VAS scores. VAS, PPI, and PRI together constitute the Short-Form McGill Pain Questionnaire (SF-MPQ), which assesses multiple dimensions of pain, including severity, type, and emotional aspects (25). Future research could further refine these assessments to better understand acupuncture is effectiveness across different pain types. Several studies have explored the mechanisms of acupuncture in treating PS-TP generally attributing its effects to the promotion of functional recovery following nerve damage (26–30). Monitoring relevant indicators and exploring the correlation between pain scores and these indicators could further elucidate the efficacy and mechanisms of acupuncture.

Recent advancements in studying the aftereffects of acupuncture highlight that these effects change over time, differing from the immediate effects targeting the spinal cord (31, 32). These aftereffects might influence the extrapyramidal system or neural circuits in the brain (33). As the cumulative aftereffects increase with more sessions, acupuncture tolerance may develop, leading to reduced analgesic effects. Thus, optimizing the frequency and duration of acupuncture sessions is crucial to balancing aftereffects, tolerance, and pain relief (34, 35). Follow-up studies showed that SF-MPQ of the treatment group remained lower than those of the control group, suggesting sustained benefits of acupuncture. However, variations in sample sizes, follow-up durations, acupoint selection, and techniques might affect the accuracy of these findings.

The minimal clinically important difference (MCID), a critical metric for evaluating the clinical significance of therapeutic effects, plays a pivotal role in both clinical research and medical practice. Currently, there is no established MCID threshold for the VAS in central neuropathic pain. Therefore, we referenced an MCID of 1.5 derived from chronic pain studies involving fibromyalgia and knee osteoarthritis (36). Compared to these conditions, PS-TP involves central nervous system damage with distinct pathophysiological mechanisms, which may result in more limited treatment-related improvements. This suggested that the MCID for PS-TP could be smaller than 1.5. Furthermore, based on the post-intervention analysis of VAS scores in this study MD was −1.87, the observed improvement did not reach the hypothesized MCID within the current treatment duration. Future studies should prioritize defining MCID thresholds specific to PS-TP and extending follow-up periods to investigate the optimal acupuncture treatment duration for this condition.

#### 4.1.2 Analgesic mechanism

Acupuncture is analgesic mechanisms include the gate control theory of pain, diffuse noxious inhibitory control theory, reduction of central nervous system excitability, and modulation of neurotransmitters and immune functions. Recent studies have linked variations in blood-related indicators with pain-related disease processes (37–41). Acupuncture is analgesic effects involve bidirectional modulation of central neurotransmitters to suppress pain transmission. Acupuncture stimulates neural impulses that involve various chemicals, cytokines, and antagonistic factors (42). Enhancing acupuncture analgesia are substances such as β-EP, serotonin (5-HT), endogenous morphine-like substances, and acetylcholine, while substances like SP, cholecystokinin octapeptide, and GABA may oppose its effects. Three studies reported changes in β-EP and SP levels (43), indicating that acupuncture may reduce β-EP levels and increase SP levels, thus alleviating PS-TP.

#### 4.1.3 Safety evaluation

Pharmacological treatments for PS-TP often have various adverse reactions, and non-pharmacological interventions lack standardized safety profiles (44, 45). Literature suggests that that acupuncture has a far lower rate of adverse events than Western treatment (46). Five studies reported adverse events, with four only detailing names and incidence rates. Two studies (Wang S. M. 2018 and Zhang, Q. X. 2021) noted the incidence and resolution time of acupuncture-induced bruising. One study (Wang M. 2014) also detailed measures taken and outcomes for adverse events. Overall, adverse events were lower in the treatment groups than the control groups, and acupuncture alone had fewer adverse events than combined acupuncture and medication treatments. This implies that acupuncture has a safety benefit for PS-TP. However, due to small sample sizes and various confounding factors, larger-scale real-world studies are needed to further investigate safety.

#### 4.1.4 Mechanisms of pain emotion

Neuroscientific research confirms that pain-related emotions are integral to the pain experience, originating from thalamic pain signals and affecting pain perception (47). Acupuncture therapy has shown effectiveness in the regulation of pain-related emotions, not only alleviating pain but also modulating negative emotions (48, 49). This dual benefit highlights a significant advantage of acupuncture in pain management.

#### 4.1.5 GRADE assessment and TSA

TSA was performed for all outcome indicators. Except for safety, the analyses were conclusive, suggesting no need for additional trials. GRADE assessment rated only total efficacy rate as moderate quality and the VAS score as low quality, while other indicators were rated very low. This rating is attributed to lack of blinding, small sample sizes, and publication bias. Future clinical studies should adhere to rigorous design and implementation standards to enhance research quality.

### 4.2 Strengths and limitations

#### 4.2.1 Strengths

The strengths of this study include: (1) Incorporation of recent, high-quality studies with larger sample sizes, allowing a more comprehensive analysis. Previous reviews lacked detailed meta-analyses on post-effects, safety, and neuropeptide levels. (2) Evaluation of acupuncture is therapeutic effects across multiple indicators, reflecting its multi-mechanism and multi-target nature. (3) Use of subgroup and sensitivity analyses to address heterogeneity. (4) Applying qualitative and quantitative methods to assess publication bias, enhancing credibility. (5) Innovative use of TSA to adjust for random errors, ensuring reliable meta-analysis results.

#### 4.2.2 Limitations

This study has limitations: (1) Variability in acupuncture protocols, including location, technique, and patient factors, affects efficacy and data accuracy (35, 50–53). (2) Given the lack of positive control drugs with established therapeutic efficacy for this disease, heterogeneous control group interventions may increase inter-study heterogeneity. (3) There is considerable variation in how side effects are reported across different studies, some studies do not provide detailed reports of adverse reactions, which impacts the comprehensive assessment of the safety of acupuncture treatment. (3) Non-standardized randomization and the lack of blinding in included studies could lead to bias. (4) Because all research was done in China, generalizability may be limited. (5) Low quality of evidence for some indicators, as assessed by GRADE, may impact result accuracy.

### 4.3 Implications for further research

This study provides a comprehensive analysis of the efficacy and safety of acupuncture for PS-TP, evaluating multiple indicators, including overall efficacy rates, scale scores, blood test results, and adverse reaction rates. To address the limitations of studies and enhance future research, several areas for improvement are suggested: (1) Sample size: Increasing sample sizes in future studies can enhance statistical power, reduce random errors, and improve the reliability of conclusions. (2) Study design: Adhering to Cochrane Handbook guidelines is crucial. Future studies should provide detailed reports on random sequence generation, allocation concealment, and blinding implementation. While blinding researchers is challenging in acupuncture studies, using sham acupuncture for the control group can blind participants. Incorporating a placebo-controlled design (acupuncture + placebo vs. sham acupuncture + medication) can help meet blinding and ethical requirements. As the field advances, it is imperative to establish standardized control protocols specifically tailored for PS-TP research. Such standardization will enhance the comparability and reliability of findings across studies, ultimately facilitating evidence-based clinical decision-making. (3) Safety evaluation: More focus is needed on safety aspects. Future research should more thoroughly document and report adverse reactions to comprehensively assess the safety of acupuncture. (4) Long-term effects: Current studies lack sufficient attention to the long-term effects of acupuncture. Future research should include multiple follow-up time points post-treatment to better understand the enduring effects and patterns of acupuncture for PS-TP. (5) Objective assessment methods: Investigate mechanisms using objective assessment tools such as blood tests, functional magnetic resonance imaging, and electroencephalography. These methods can provide deeper insights into the mechanisms of acupuncture. (6) Pain emotion assessment: There should be an increased emphasis on evaluating pain emotion indicators to more accurately assess the comprehensive efficacy of acupuncture therapy.

Addressing these areas will contribute to a more thorough understanding of acupuncture’s effectiveness and safety for PS-TP.

## 5 Conclusion

This study demonstrates that acupuncture is effective in improving clinical outcomes and reducing pain scores for PS-TP, with the added benefit of reducing β-EP levels and increasing SP levels. It offers certain advantages in reducing pain scores long-term, without significant adverse reactions observed. While the evidence supports acupuncture as an effective treatment for PS-TP, the small sample size and variability in study designs call for further research. Continued exploration through RCTs is necessary to provide more substantial evidence for clinical practice.

## Data Availability

The original contributions presented in the study are included in the article/supplementary material, further inquiries can be directed to the corresponding authors.
